# Performance of commercial growing-finishing pigs fed supplemental isoquinoline alkaloids: a statistical process control analysis

**DOI:** 10.1186/s40813-023-00311-3

**Published:** 2023-07-05

**Authors:** V. Artuso-Ponte, T. Steiner, F. Neher, Emilio L. Cano, A. Morillo-Alujas

**Affiliations:** 1 Phytobiotics Futterzusatzstoffe GmbH, Eltville, Germany; 2 Data Science Laboratory, Rey Juan Carlos University, Móstoles, Madrid Spain; 3 Tests and Trials, Monzón, Huesca, Spain

**Keywords:** SPC, Grow-finish pigs, Performance, Plant-based feed additive

## Abstract

**Background:**

Statistical Process Control (SPC) is a powerful statistical tool that can be used in animal production to evaluate the evolution of production parameters overtime in response to the implementation of a specific strategy. The aim of this study was to evaluate the effect of supplementing growing-finishing pigs with isoquinoline alkaloids (IQ) on growth performance parameters by using the SPC method. IQ are natural secondary plant metabolites which have been extensively investigated in food animals due to their efficacy in supporting growth performance and the overall health status. Performance parameters and medication usage were collected from 1,283,880 growing-finishing pigs fed the same basal diet, 147,727 of which were supplemented with IQ from day 70 of life until slaughter.

**Results:**

Supplementation with IQ improved feed conversion ratio, while feed intake and daily gain were maintained.

**Conclusion:**

SPC methods are useful statistical tools to evaluate the effect of using a new feed additive in the feed of pigs on growth performance at a commercial level. Additionally, IQ supplementation improved growth performance and it can be considered as a good strategy to reduce feed conversion in growing-finishing pigs.

## Introduction

Many factors influence the efficiency of animal production systems, including the implementation of new or different feeding strategies to optimize growth performance. Therefore, it is essential to constantly monitor performance, although this is usually a complex task due to the natural variation of these processes.

Statistical Process Control (SPC) is a statistical method that can be used to monitor performance and detect significant changes due to unplanned or planned interventions, such as the occurrence of a disease outbreak or the application of a new vaccination program. SPC tools have been used in manufacturing industries since the 1920s and in animal production [7]. For example, SPC methods were used in pre-weaned calves to monitor their feeding behavior and predict the occurrence of a sick calf [11]. Most recently, SPC methods were successfully used to evaluate the variation of bulk tank milk somatic cell count in small dairy farms to determine their milk quality [16]. However, to our knowledge, there are not publications about the use of SPC methods to evaluate growth performance in growing-finishing pigs. Isoquinoline Alkaloids (IQ including sanguinarine, chelerythrine, protopine and allocryptopine are usually obtained from Papaveraceae plants and have been used in farm animals and aquaculture. IQ have shown anti-inflammatory, immune-modulatory and stress-relieving effects and have been successfully included in alternative strategies to the use of antibiotic growth promoters [2, 10]. IQ have been used in production animals to improve growth performance and enhance digestibility of nutrients [18]. Supplementation in weaning pigs with IQ has shown to improve the intestinal barrier function and reduce the diarrhea score [13]. Additionally, IQ-fed finishing pigs have demonstrated lower levels of cortisol after transportation to slaughter, which was positively correlated to a decrease in *Salmonella* shedding, indicating that IQ supplementation reduces stress in pigs and the negative impact on the gastrointestinal tract [2]. In sows, it has been recently demonstrated that IQ supplementation during lactation reduced body weight loss and increased colostrum quality [1]. De Souza Massei et al. [6] have recently investigated the effect of IQ supplementation on performance parameters, carcass characteristics and health in growing and finishing pigs under experimental conditions. However, published studies on the effect of IQ supplementation on performance parameters of growing and finishing pigs under field conditions are not available. Moreover, all the afore-mentioned studies were carried out under experimental conditions, characterized by a high genetic, feed and health status variation and hence not necessarily representing commercial production conditions. Therefore, external validity of the experimental studies might be compromised.

The aim of the study was to evaluate the effect of supplementing commercial growing-finishing pigs with plant-based IQ on production performance by using SPC methods.

## Materials and methods

The study was carried out in a commercial swine integration in Spain. The pigs included in the study originated from 20 sow farms owned or contracted by the integrator. Historical control (calibration) data was compiled from January 2020 to June 2020. During this period, all animals received a standard commercial diet based on barley, wheat, corn, and soybean meal. All diets were manufactured in and delivered from the same feed mill. During the supplementation period, which started in June 2020 and lasted until February 2021, all pigs were fed the same standard diet supplemented with 1 kg/t feed of a plant-based IQ product (Phytobiotics Futterzusatzstoffe GmbH, Eltville, Germany), from day 70 of life until slaughter. After the study, a post-supplementation follow-up data gathering was performed, i.e., production variables were recorded whilst the regular, calibration-period diet, was delivered to the animals. This post-supplementation period lasted from January 2021 until March 2022.

An SPC approach was performed [4]. SPC is a statistical technique that aims at detecting change in processes by monitoring the variation of a given quality characteristic against the in-control process. Thus, when non-random variation according to the in-control process (Phase I) occurs, then there is evidence that the process has changed and the assignable causes of variation should be found and eliminated, i.e., an unplanned intervention is needed. Planned interventions arise when SPC is used to confirm that a given change in the process, e.g., a supplemented diet, leads to the desired change in the process, in this case better production performance. For this type of studies, the in-control process corresponds to SPC Phase I, historical pre-supplementation period, whereas Phase II corresponds to the supplementation period, after the change was introduced in the production process. Sometimes, like in the case at hand, there could be also a Phase III when the process goes back to the state before the intervention. In this case, this Phase III corresponds with the post-supplementation period.

Data from 188 batches representing 458,031 pigs was used as control (before IQ; SPC Phase I); a total of 56 batches representing 147,727 pigs were included in the supplementation period (SPC Phase II), whereas the data from 268 batches with a total of 678,122 pigs was used as control post supplementation (after IQ; SPC Phase III). All farms included in the study belonged to the same integration, health, genetic and nutritional conditions were very similar across farms. Nevertheless, all these variables, which may influence the results were included in the analysis.

Data recorded during the three periods, historical control, supplementation and post supplementation included feed conversion ratio (FCR), average daily gain (ADG, g/d), average daily feed intake (ADFI, g/d), cost of medicines (Euro/pig), runts (%) and mortality rate (%). Hierarchical Clustering on Principal Components (HCPC) analysis was performed using the nutritional scheme dataset, resulting inthree nutritional clusters that were used to analyze the evolution of the growth performance considering the changes in nutrition. Differences in nutrition were due to differences in Standardized Digestible Lysine (SID Lys, %) and the level of Net Energy in kcal/kg (NE) between the feed batches used during the study period (Table 1).

**Table 1:** Characteristics of nutritional clusters

	Net energy (kcal/kg)	SID lys %
Nutritional cluster 1	2456	0.799
Nutritional cluster 2	2451	0.816
Nutritional cluster 3	2451	0.821

The batches that did not fit into a defined nutritional cluster were added to a fourth cluster

Furthermore, a k-means Clustering Analysis was performed with the pam algorithm, resulting on three health clusters that were used to analyze the evolution of the study taking into account the health status of the pigs by origin, as some of the animals were purchased outside the integration. The health status was characterized based on PRRSv, *Actinobacillus pleuropneumoniae* (APP), *Mycoplasma hyopneumoniae* (Myo), *Lawsonia intracelullaris* and *Streptococcus suis* status (Table 2).

**Table 2:** Characteristics of health clusters

	*Streptococcus* and APP	PRRS	*Mycoplasma hyopneumoniae*	Lawsonia
Health cluster 1	Positive	Negative	Negative	Negative
Health cluster 2	Negative	Positive	Positive	Negative
Health cluster 3	Negative	Negative	Negative	Positive

The batches that did not fit into a defined health cluster were added to a fourth cluster

SPC tools were used to monitor the previously described performance parameters. The R statistical software and programming language [15] and the qcc R package [19] were used for the computations and visualization. Cumulative sum (CUSUM) control charts were obtained for all parameters and for each nutritional and health cluster to show the evolution or the changes of each parameter. The analysis of this type of charts demonstrates how the evaluated parameters change over time and the SPC detects if the change was likely caused by the supplementation with IQ.

Furthermore, boxplot charts were constructed for all parameters to evaluate the dispersion of the data in all, control, supplementation, and post supplementation periods. In addition, the effect of the seasonality was considered, as some parameters changed in the different months. Therefore, boxplots were constructed by group, cluster and for each month of the study period. Finally, analysis of variance (ANOVA) was performed for all parameters, including post-hoc Tukey’s pairwise contrasts. A p-value ≤ 0.05 was considered statistically significant and a trend was determined at a *p*-value ≤ 0.10. Effect sizes ${\eta }^{2}$ of the ANOVA Type II tests were also computed and labelled following Field’s [8] recommendations, i.e., < 0.01 small effect, 0.06 medium effect and 0.14 large effect.

## Results

Overall means and standard deviation for growth performance, mortality, % runts and medication data are presented in Table 3.

**Table 3:** Overall means and standard deviation (SD) for growth performance, cost of medicine, mortality and runts

	Before IQ		IQ supplementation		After IQ		*p*-Value
	Mean	SD	Mean	SD	Mean	SD	
ADFI (g/d)	2118.94	290.28	2036.46	282.39	2103.02	273.29	0.11
ADG (g/d)	852.17	85.99	851.34	88.44	861.30	82.30	0.44
FCR	2.55^a^	0.15	2.45^b^	0.12	2.51^a^	0.14	0.007
Cost of medicine (euro/pig)	1.22	0.86	1.07	0.76	1.17	0.80	0.41
Mortality (%)	4.18	2.57	4.49	3.09	4.21	2.75	0.52
Runts (%)	1.07	0.77	1.15	1.07	0.93	0.78	0.16

The Tukey’s test was performed to detect differences between the study periods

*Before IQ* Before supplementation period; *IQ supplementation* supplementation period with isoquinoline alkaloids; *After IQ* after supplementation period

^a,b^*p* ≤ 0.05

### Growth performance

Overall, ADFI and ADG were not different between the three study periods (before IQ, IQ supplementation and after IQ; *p* ˃ 0.05). FCR was significantly lower during the period where pigs were supplemented with IQ, as compared to both periods, before and after IQ supplementation (*p* = 0.007).

Means and standard deviations for each nutritional and health cluster with or without IQ supplementation are presented in Table 4.

**Table 4:** Means and standard deviation for growth performance, cost of medicine, mortality and runts for each nutritional (N) and health (H) cluster

	FCR		ADG (g/d)		ADFI (g/d)		Mortality (%)		Runt (%)		Medication cost (Euro/pig)	
	IQ	CON	IQ	CON	IQ	CON	IQ	CON	IQ	CON	IQ	CON
N 1	2.5^a^ (0.2)	2.6^b^ (0.1)	850.5 (75.4)	857.7 (90.5)	2117.1^b^ (271.3)	2168.6^a^ (282.6)	5.8 (3.3)	4.8 (2.4)	1 (1.0)	1.2 (0.7)	1.1 (0.6)	1.8 (1.4)
N 2		2.6 (0.2)		858.2 (74.2)		2154.2 (258.4)		4 (2.5)		1 (0.7)		1.1 (0.8)
N 3	2.4^a^ (0.1)	2.5^b^ (0.1)	877.5^a^ (74.1)	844.4^b^ (96.0)	2112^a^ (240.1)	2068.3^b^ (318.0)	3.5 (2.3)	4.3 (2.7)	1 (1.1)	1.1 (0.9)	1 (0.7)	1.2 (0.7)
N 4	2.3 (0.1)		787.5 (37.6)		1866.2 (165.6)		10.9 (4.6)		2.2 (0.1)		1.4 (0.7)	
H 1	2.4 (0.1)	2.5 (0.2)	893.2 (67.6)	835.1 (91.4)	2185.1 (219.8)	2033.3 (340.6)	4.6 (2.1)	6.7 (3.8)	0.9 (1.0)	1.7 (0.6)	2.5 (0.5)	1.7 (0.9)
H 2	2.4 (0.1)	2.4 (0.1)	800.7 (75.5)	733.5 (40.6)	1816 (155.2)	1737.8 (124.1)	6.2 (5.2)	5 (2.4)	1.2 (2.2)	1 (0.8)	1.3 (1.2)	2 (1.3)
H 3	2.5 (0.1)	2.6 (0.2)	892.5 (62.1)	880 (61.8)	2159.7 (210.6)	2205.6 (218.7)	3.2 (1.7)	3.6 (1.9)	1 (0.9)	0.9 (0.6)	0.8 (0.4)	1.1 (0.7)
H 4	2.5 (0.1)	2.6 (0.1)	844.7 (82.6)	866.6 (86.4)	2073.8 (274.0)	2180.3 (278.9)	5.2 (3.7)	3.7 (2.1)	1.2 (1.0)	1.1 (0.9)	1 (0.7)	1 (0.6)

The Tukey’s test was performed to detect differences between the study periods

*FCR* feed conversion ratio; *ADG* average daily gain; *ADFI* average daily feed intake; *IQ* isoquinoline alkaloid supplementation period; *CON* without isoquinoline alkaloids supplementation period. Nutritional cluster 2 (N 2) and 4 (N 4) were not identified during the isoquinoline alkaloids supplementation period and the period without isoquinoline alkaloids supplementation, respectively

^a,b^*p* ≤ 0.05

When comparisons were made by nutritional or health clusters it was found that FCR was significantly lower in the IQ supplementation period in nutritional cluster 1, (*p* = 0.004), nutritional cluster 3 (*p* = 0.006), and tended to be lower in health cluster 3 (*p* = 0.07). ADFI was higher in nutritional cluster 1 for the periods without IQ supplementation as compared to the period with IQ in the feed (*p* = 0.014). ADG and ADFI were higher in nutritional cluster 3 for the periods with IQ supplementation (*p* = 0.001 and p < 0.001, respectively). ADFI tended to be higher during the periods without IQ supplementation in health cluster 4 (*p* = 0.07).

### Cost of medicine, mortality and runts

The cost of medicine, which included all types of water and injectable medications, was not different between the periods with or without IQ supplementation (*p* = 0.41).

Overall mortality and runts were not different between the periods with and without IQ supplementation (p = 0.52 and p = 0.16, respectively).

### CUSUM charts

**Fig. 1:**
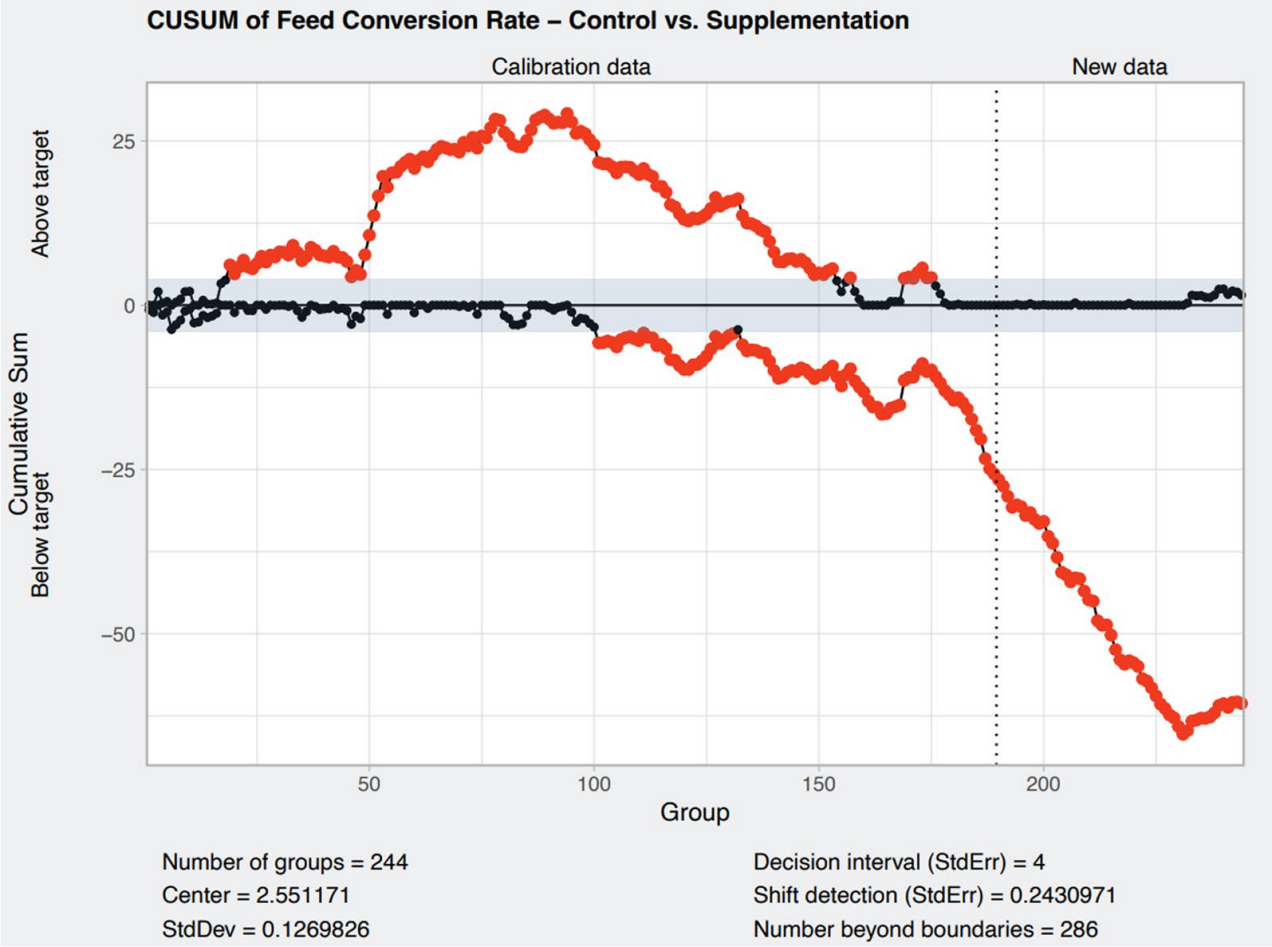
Cumulative sum control chart for FCR. Calibration data included feed conversion ratio data from all batches of pigs produced in all the farms enrolled in the study without isoquinoline alkaloids supplementation (before isoquinoline alkaloid supplementation). New data include the feed conversion ratio from all batches of pigs produced during the period where isoquinoline alkaloids were included in the feed. Red dots represent values outside the set limits and indicate a significant shift in feed conversion ratio

**Fig. 2:**
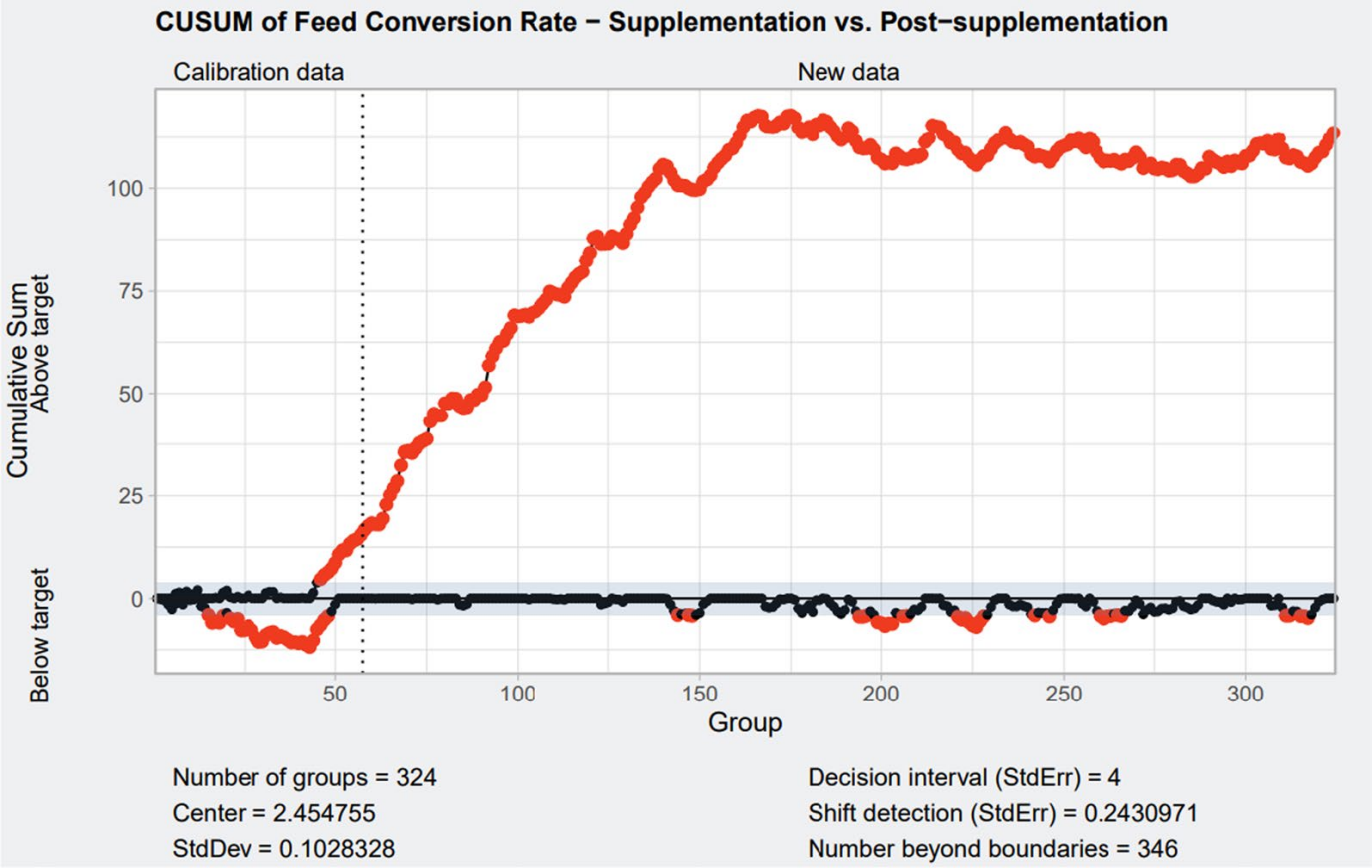
Cumulative sum control chart for feed conversion ratio. Calibration data included the feed conversion ratio data from all batches of pigs produced in all the farms enrolled in the study with isoquinoline alkaloids supplementation (during isoquinoline alkaloid supplementation). New data include feed conversion ratio from all batches of pigs produced after isoquinoline alkaloids supplementation. Red dots represent values outside the set limits and indicate a significant shift in feed conversion ratio

**Fig. 3:**
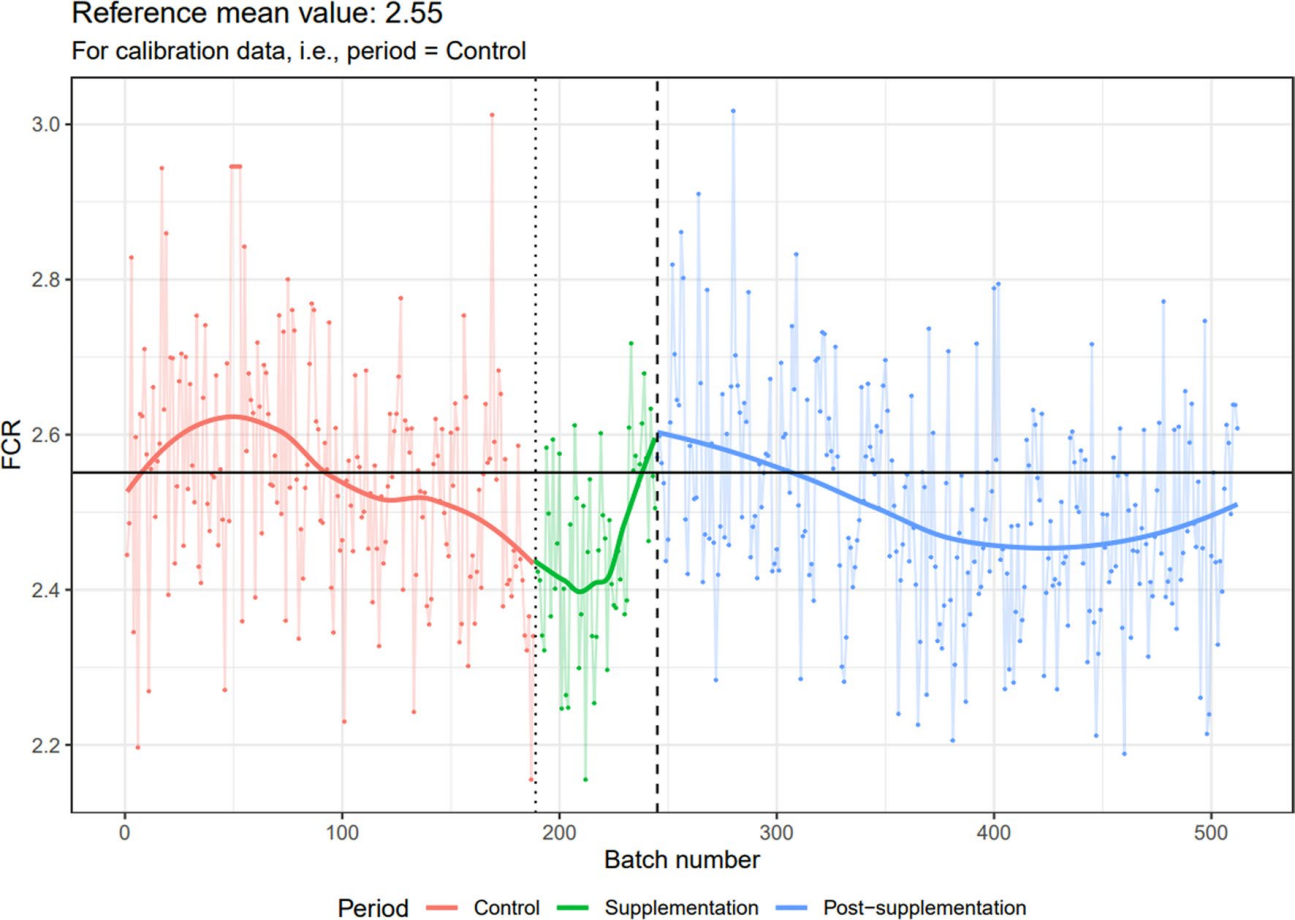
Evolution of feed conversion ratio data during the three experimental periods: before isoquinoline alkaloids, isoquinoline alkaloid supplementation and after isoquinoline alkaloid supplementation. Solid lines represent trend curves fitted with local polynomial regression

The CUSUM control charts are designed in order to detect a change of ~ 0.24 standard deviations, i.e., to achieve a value of ~ 2.31 for FCR. Cumulative sums of positive and negative differences with the mean of the calibration period are computed and represented in the chart. A positive shift results in an upward trend of the upper line, and a negative shift results in a downward trend of the lower line. The size of the shift is measured with the slope of the trend. If the trend keeps on, the change is consolidated. We can see that after introducing isoquinoline alkaloids in the feed (Fig. 1) a shift is detected, and it is sustained all over the period. Furthermore, using the supplementation period as calibration data and the post supplementation period (Fig. 2) as new data, we can clearly see how an upper shift is detected and kept for a number of batches. At some point the trend goes down again, but in a small amount, without reaching the values of the supplementation period. Figure 3 shows the overall evolution of the FCR that reflects the three phases evolution in the scale of the variable.

## Discussion

The primary findings in this study are the following: (1) SPC methods are useful tools to evaluate growth performance in pigs under field conditions and to determine if and where significant shifts occur in response to the inclusion of a feed additive; and (2) the use of IQ improved FCR in growing-finishing pigs when supplemented from day 70 of life until slaughter.

SPC methods are useful statistical tools to monitor growth performance in animal production, although they are not widely used to determine how growth performance parameters change in response to the inclusion or use of a new strategy, such as the addition of a feed additive [7]. Several studies have been conducted in pigs under experimental conditions [3, 9, 17, 18]. However, this is the first study evaluating the effect of IQ supplementation by using SPC methods under field conditions. Previous research studies carried out to evaluate the effect of IQ supplementation have been carried out in research facilities, which not always represent the real conditions the animals are subjected to under commercial conditions. Although randomized control designs are preferred to determine causation, the results cannot always be extrapolated to the general population as environmental, nutritional, management, health, genetics conditions can vary between farms and even within farms. On the other hand, SPC methods can be used to effectively monitor growth performance in pigs under commercial conditions and to determine if the observed changes in performance can be related to the use of new interventions [7].

The results of this study showed that the overall FCR shifted down after the inclusion of IQ, whereas it when back up after IQ were taken off the feed. At this moment, there is not published data showing the effect of IQ supplementation in growing-finishing pigs on growth performance under commercial conditions. In the present study, FCR was improved by 3.9% and 2.4% as compared to the period before and after IQ supplementation, respectively, which is in accordance to the improvement ranges reported previously in post weaning piglets [3, 9]. IQ have demonstrated to improve nutrient digestibility, probably due to their anti-inflammatory effect in the gut, which improves intestinal barrier function and the integrity of the gut mucosa [13, 18]. Furthermore, IQ supplementation has demonstrated to reduce salivary cortisol in growing-finishing pigs after transportation to the slaughterhouse, thus modulating stress response [2]. Additionally, IQ supplemented growing pigs have shown a reduced intestinal permeability [17]. IQ, specifically sanguinarine has been demonstrated to inhibit the activation of the transcription factor NF-kB, which is known to play an essential role in intestinal inflammation [5, 12]. Therefore, intestinal inflammation is modulated, which improves the intestinal barrier function, promoting a better nutrient digestion and absorption and minimizing the impact of intestinal disorders. During the finishing period, pigs are subjected to several stress conditions such as overcrowding, feed deprivation, low air quality, among others, which may compromise intestinal health, increasing intestinal permeability and allowing the entrance of pathogens and macromolecules, which negatively influence growth performance and the health status of the animals [14].

In conclusion, IQ supplementation effectively improved FCR in growing-finishing pigs under field conditions. Moreover, SPC tools are useful to monitor growth performance and evaluate changes in response to new interventions at a lower cost and high statistical power.

## Data Availability

Not applicable.
